# Hypothalamic kinin B1 receptor mediates orexin system hyperactivity in neurogenic hypertension

**DOI:** 10.1038/s41598-021-00522-0

**Published:** 2021-10-26

**Authors:** Rohan Umesh Parekh, Acacia White, Korin E. Leffler, Vinicia C. Biancardi, Jeffrey B. Eells, Abdel A. Abdel-Rahman, Srinivas Sriramula

**Affiliations:** 1 Department of Pharmacology and Toxicology, Brody School of Medicine at East Carolina University, 600 Moye Blvd, Greenville, NC 27834 USA; 2 Department of Anatomy, Physiology and Pharmacology, College of Veterinary Medicine, Auburn University, Auburn, AL USA; 3 Center for Neuroscience Initiative, Auburn University, Auburn, AL USA; 4 4Department of Anatomy and Cell Biology, Brody School of Medicine at East, Carolina University, Greenville, NC 27834 USA

**Keywords:** Neuroscience, Hypertension

## Abstract

Brain orexin system hyperactivity contributes to neurogenic hypertension. We previously reported upregulated neuronal kinin B1 receptor (B1R) expression in hypertension. However, the role of central B1R activation on the orexin system in neurogenic hypertension has not been examined. We hypothesized that kinin B1R contributes to hypertension via upregulation of brain orexin-arginine vasopressin signaling. We utilized deoxycorticosterone acetate (DOCA)-salt hypertension model in wild-type (WT) and B1R knockout (B1RKO) mice. In WT mice, DOCA-salt-treatment increased gene and protein expression of orexin A, orexin receptor 1, and orexin receptor 2 in the hypothalamic paraventricular nucleus and these effects were attenuated in B1RKO mice. Furthermore, DOCA-salt- treatment increased plasma arginine vasopressin levels in WT mice, but not in B1RKO mice. Cultured primary hypothalamic neurons expressed orexin A and orexin receptor 1. B1R specific agonist (LDABK) stimulation of primary neurons increased B1R protein expression, which was abrogated by B1R selective antagonist R715 but not by the dual orexin receptor antagonist, ACT 462206, suggesting that B1R is upstream of the orexin system. These data provide novel evidence that B1R blockade blunts orexin hyperactivity and constitutes a potential therapeutic target for the treatment of salt-sensitive hypertension.

## Introduction

Hypertension remains the single most important contributor to the global burden of disease and mortality as diagnoses of high blood pressure are predicted to rise in all regions of the world^1^. Epidemiological data shows that the incidences of hypertension worldwide will continue increasing over the next decade, with three quarters of men and women over 60 years of age diagnosed with hypertension, as the global population as a whole is growing and aging. The etiology of hypertension involves complex interactions of environmental and pathophysiological factors across multiple organ systems and a genetic component^2^. High blood pressure predisposes to cardiovascular and renal diseases, cognitive and neuroanatomical functional disorders. Preventative strategies are urgently desired, and current hypertensive therapeutics need optimization for appropriate management and reduced long-term mortality. While lifestyle modifications and current pharmacological therapies are effective in lowering blood pressure, approximately 12–18% of diagnosed patients suffer from uncontrolled, refractory hypertension^2^. Additionally, certain patient populations, such as the African American demographic, are diagnosed at a significantly higher rate than other comparative demographics and have one of the highest levels of refractory hypertension cases^2,3^.

The rising incidence of hypertension, coupled with a high rate of diagnosed, yet uncontrolled cases, underscores the need for mechanistic research studies for developing novel pharmacotherapeutics. Most of the clinical therapies currently utilized focus on targeting the overactivation of the renin-angiotensin system in the periphery, highlighting the need for pharmacotherapeutics targeting novel centrally acting mechanisms as alternative therapies in patients that do not respond to the renin-angiotensin targeted therapeutics. Recently, mounting evidence has highlighted the significant role of the orexin system in centrally mediated mechanisms that underlie neurogenic hypertensive state^4–6^.

Orexin, also known as hypocretin, a neuropeptide produced by hypothalamic neurons, mediates a vast array of physiological functions, including enhancing central sympathetic activity and elevating blood pressure^4,6^. The two neuropeptides orexin A and orexin B exert their actions via two G-protein coupled receptors, the orexin receptor 1 (OX1R) and orexin receptor 2 (OX2R)^7^. Studies in several hypertensive animal models showed that increased orexin system activity contributes to elevated blood pressure and increased sympathetic nerve activity (SNA)^6,8–10^. Upregulation of OX1R by orexin A increases the firing of the paraventricular nucleus neurons, augments SNA, and contributes to hypertension in genetic hypertensive rat models^11^. Administration of a blocker of the orexin system ameliorates hypertension and overactive SNA^4,6,12^. Further, orexin signaling in the paraventricular nucleus appears critical for the onset and maintenance of hypertension via arginine vasopressin (AVP) upregulation in the DOCA-salt model of hypertension^12^. The orexin system enhances AVP release, and studies have shown that elevated AVP release is necessary for a hypertensive state in a neurogenic animal model of hypertension^13^. Interestingly, in African American patients, the elderly and patients with congestive heart failure or chronic renal failure all exhibit AVP-dependent hemodynamic changes, and these populations exhibit low levels of circulating renin^13,14^. These parallels suggest that the high level of uncontrolled neurogenic hypertension in these patient groups may be better controlled with a pharmacotherapeutic targeting the central orexin and AVP hyperactivities.

The kinins are a family of vasoactive proinflammatory peptides that mediate copious physiological actions related to cardiovascular homeostasis and inflammatory responses^15^. Clinical and experimental studies have suggested a significant role for kinins and their receptors in the maintenance of normotension and the development of hypertension. The physiological effects of kinins are mediated by two G-protein coupled receptor subtypes, kinin B1 (B1R) and B2 (B2R) receptors. The nature of these two receptors is distinct, with B2R thought to be constitutively expressed and B1R expression being upregulated in the presence of cytokines^15^. While substantial evidence indicates that the orexin system is heavily implicated in blood pressure regulation, the role of neuronal B1R in the orexin system has not been previously investigated. The B1R is a novel target for the treatment of neurogenic hypertension because it mediates neuroinflammation and immune cell infiltration, which contribute to hypertension^16^. Animal studies that support B1R involvement in the pathogenesis of hypertension showed increased B1R mRNA expression^15,17^ and increased densities of the B1R-binding sites^18^ in hypertension models. Additionally, upregulated B1R and increased blood pressure are observed in both rat and mouse models, and blockade of B1R mitigates blood pressure elevation in these models^15,19^. We have previously reported higher B1R expression and brain AVP levels in hypertensive mice^15^, yet, until the present study, the interplay between B1R and the orexin system, particularly in neurogenic hypertension, has remained unknown. Thus, in the present study, we investigated if kinin B1R contributes to hypertension via upregulating the orexin-AVP signaling in the brain. Additionally, we examined the role of B1R in the modulation of the orexin system in both in vivo (DOCA-salt paradigm) and in vitro (in primary neuron culture). Further, we hypothesize that knockdown of B1R in a mouse model will mitigate neurogenic hypertension, at least partly, by reducing the pro-hypertensive hypothalamic orexin system.

## Methods

### Animals

All mice were housed in a temperature- and humidity-controlled facility under a 12-h dark/light cycle, fed standard mouse chow and water ad libitum. All experiments were conducted on adult male mice (12–16 weeks old). Kinin B1 receptor knockout (B1RKO) mice were a generous gift from Dr. Michael Bader (Charité Hospital, Berlin, Germany). They originated from 10 generations of backcrossing of an initially mixed genetic background (129/Sc and C57Bl/6) with C57Bl/6 mice^15,20^. The B1RKO mice used for experiments were generated by mating homozygous mice and confirmed with genotyping as described previously^15^. To maintain a stable inbred background, the B1RKO mice backcrossed to the standard C57Bl/6NJ strain about every 10 generations. Wildtype (WT) C57Bl/6NJ mice were purchased from the Jackson Laboratory. All animal studies were approved by the East Carolina University Animal Care and Use Committee (AUP #W254) and were performed in accordance with the National Institutes of Health Guidelines for the Care and Use of Laboratory Animals and in compliance with the ARRIVE guidelines.

### DOCA-salt hypertension model

Mice were anesthetized with isoflurane (2%, oxygen flow 1 L/min) and placed on a heating pad to maintain body temperature. Post-operative care included a sustained-release Buprenorphine SR injection to relieve pain at the end of the surgery (0.05 mg/Kg, sc). The mice underwent uni-nephrectomy. An incision was made on the skin in the retroperitoneal region, and the right kidney was removed. Mice were then randomly divided into 4 groups (n = 12/group) and implanted subcutaneously either with a DOCA-silicone sheet (DOCA group, DOCA:silicone = 1:3; DOCA 1 mg/g body weight) or an empty silicone sheet (Sham group)^21,22^. The mice receiving DOCA were switched to 1% NaCl in drinking water, and the sham mice received autoclaved tap water. At the end of the protocol, mice were placed under heavy anesthesia using 5% isoflurane, and euthanized by decapitation. The blood was collected for plasma. The brains were collected and stored at − 80 °C until used.

## Hypothalamic primary neurons culture

Primary neurons were cultured from neonatal, or 1-day-old mice pups as described^23,24^. Briefly, mouse pups were anesthetized with isoflurane (4%) in an oxygen flow (1 L/min) before decapitation and brains were collected in ice-cold Hank’s balanced salt solution (HBSS) (14,175–079 Gibco, New York, NY, USA). Hypothalamic tissue was dissected, collected, and minced into small pieces using a sterile blade. Minced tissue was transferred into a 15 mL conical tube, washed with HBSS, then incubated with HBSS containing 1% trypsin (T1426 Sigma-Aldrich, St. Louis, MO, USA) and 1.5 kU/mL DNaseI (D5025 Sigma-Aldrich), digested for 10 min at 37 °C. Then, the tissue was washed with HBSS with 20% FBS twice, followed by washing twice with HBSS. The tissue was further triturated in HBSS containing DNase I, using a pipette with 1 mL pipette tip (6 times) and then with a 200 μL pipette tip (6 times) attached to a 10 mL serological pipette. Following the disassociation, the cells were spun down by centrifugation and resuspended in complete Neurobasal culture medium supplemented with 2% B27, 0.5 mM GlutaMax and penicillin/streptomycin (100 U/mL and 100 μg/mL, respectively) (Gibco). Dissociated neurons were then plated at a density of 50,000 cells per ml onto poly-L-lysine-coated 6-well plates. The neurons were grown in a humidified atmosphere of 5% CO_2_–95% air at 37 °C. After 24 h, additional fresh medium was added to the cells. On the fourth day, cytosine arabinofuranoside (Ara-C, 2 µM, C1768 Sigma-Aldrich) was added to the neuronal cultures to arrest the growth of non-neuronal cells. Hypothalamic neuronal cultures were previously validated using immunofluorescence labeling with a neuron-specific cytoskeletal marker, MAP2 (microtubule associated protein 2), and a glial cell-specific marker, GFAP (glial fibrillary acidic protein)^23^. The neurons cultured with Ara-C treatment showed predominantly neuronal population, demonstrating numerous processes and discrete cellular morphology of neurons with cell–cell interaction^23,24^. Hypothalamic primary neurons were cultured for at least 10 days and then used for further experiments. The treatment durations and doses of Lys-des-Arg-BK^9^ (LDABK, #3225, Tocris, 300 nM), R715 (#3407, Tocris, 10 μM), Orexin A (#06,012, Sigma, 300 nM), and ACT 462,206 (#5319, Tocris, 10 μM) are based on our preliminary studies and published literature^24–27^.

### Immunohistochemistry

Immunofluorescence detection of B1R, OX1R, OX2R, and Orexin A protein expression was done in the PVN of mice after 3 weeks of Sham or DOCA-salt treatment. Anaesthetized mice were perfused transcardially with 50 ml of ice-cold saline followed by 4% paraformaldehyde in PBS (0.01 M, pH 7.4) for 10 min as described previously^15,21,22^. The brains were removed, post fixed in 4% paraformaldehyde in PBS (0.01 M, pH 7.4) for 24 h and then placed in 30% sucrose in PBS solution for 72 h. The primary hypothalamic neuronal cells on glass cover slips and free floating mouse brain sections (30 μm, coronal) were washed with PBS and then blocked with 5% donkey serum in 1 × PBS containing 0.2% Tween-20 for 1 h, and then incubated with appropriate primary antibodies: B1R (#ABR-011, lot An-01, Alomone labs, 1:500 dilution), OX1R (#AOR-001, lot AOR001 AN0202, Alomone labs, 1:500 dilution), OX2R (#AOR-002, lot An-01, Alomone labs, 1:250 dilution), Orexin A (ab6214, lot GR291466-7, abcam, 1:500 dilution), Vasopressin (VP, #T-5048, lot A17901, BMA biomedicals, 1:15,000 dilution, used as anatomical marker) for 24 h at 4 °C. Sections were washed with PBS + 0.3% Tween-20 and incubated with appropriate Alexa flour conjugated secondary antibodies (Life Technologies, 1:1000 dilution) for 1 h at room temperature, followed by DAPI nuclear stain. Sections were mounted with ProLong Diamond Anti-Fade Mount (Invitrogen). Primary antibody omission control experiments for nonspecific binding of the secondary antibody was performed. Images were captured using an Echo Revolve Microscope or a Nikon Eclipse TE2000-E inverted microscope coupled to a Nikon A1 confocal laser.

### Quantitative real time PCR

Brains were collected from each mouse at the end of the study and were frozen on dry ice. The entire brain was later embedded in tissue freezing medium (tissue-tek O.C.T. Compound, Sakura Finetek). In a cryostat, specific brain nuclei (paraventricular nucleus) were isolated using a brain punch kit (Stoelting) according to coordinates of the Paxinos and Franklin’s mouse brain atlas. Total RNA was isolated from PVN punches using RNeasy plus micro kit with genomic DNA eliminator spin columns (Qiagen). Real Time PCR amplification reactions were performed with Power SYBR Green RNA-to-CT one-step Kit (Applied Biosystems) using a QuantStudio 6 Flex real time PCR machine (Applied Biosystems). Data were normalized to β-actin expression by the 2^−(∆∆CT)^ comparative method and expressed as a fold change compared to WT sham control.

### Protein analysis by western blot

Western blots were performed on mouse brain hypothalamic and primary hypothalamic neuronal homogenates, as described previously^24^. Tissue and neuron samples were homogenized in 1× lysis buffer containing protease and phosphatase inhibitors cocktail (Roche) and incubated on ice for 15 min. Lysates were cleared by centrifugation at 12,000×*g* and 4 °C for 15 min. After determining protein concentration using BCA protein assay kit (Thermo Fisher/Pierce), 15 µg of protein lysates were mixed with Laemmli buffer, heated at 95 °C for 5 min, and cooled on ice for 3 min. The samples were resolved on 4–15% or Any KD Mini-PROTEAN TGX gels (Bio-Rad) under reducing conditions and blotted on to PVDF membranes using Trans-Blot Turbo Transfer system (Bio-Rad). Membranes were blocked with Intercept-TBS blocking buffer (Licor) and immunoblotted overnight at 4 °C with validated antibodies against B1R (1:250, #ABR-011, Alomone labs), Orexin 1 (1:1000, #AOR-001, Alomone labs) and Orexin 2 (1:500, #AOR-002, Alomone labs). After washing, the membranes were incubated with IRDye secondary antibodies and imaged using Odyssey CLx imaging system (Licor). The blots were reprobed with GAPDH (1:1000, #MAB374, Millipore Sigma) to confirm equal loading. The density of protein bands was quantitatively analyzed by ImageJ software (NIH) and expressed as a relative ratio against the loading control.

### Plasma AVP measurement

Plasma AVP levels were measured from mouse plasma using a fluorescent enzyme immunoassay kit (FEK-065-07, Phoenix Pharmaceuticals). Peptide was extracted from plasma using Sep-Pak C18 columns (Waters Corporation). The eluted fractions were dried using a vacuum centrifuge, re-suspended in assay buffer, and used for enzyme immunoassay. The sensitivity of the assay kit was 11.2 pg/ml and linear range was 11.2–417 pg/ml.

### Statistical analysis

Data are presented as mean ± SEM. Data were analyzed by one-way or two-way ANOVA followed by Bonferroni posthoc tests for multiple comparisons between means, as appropriate. Statistical comparisons were performed using Prism 7 (GraphPad Software). Differences were considered statistically significant at *P* < 0.05.

## Results

### B1R knockdown attenuates orexin A overexpression in the PVN of DOCA-salt treated mice

**Figure 1:**
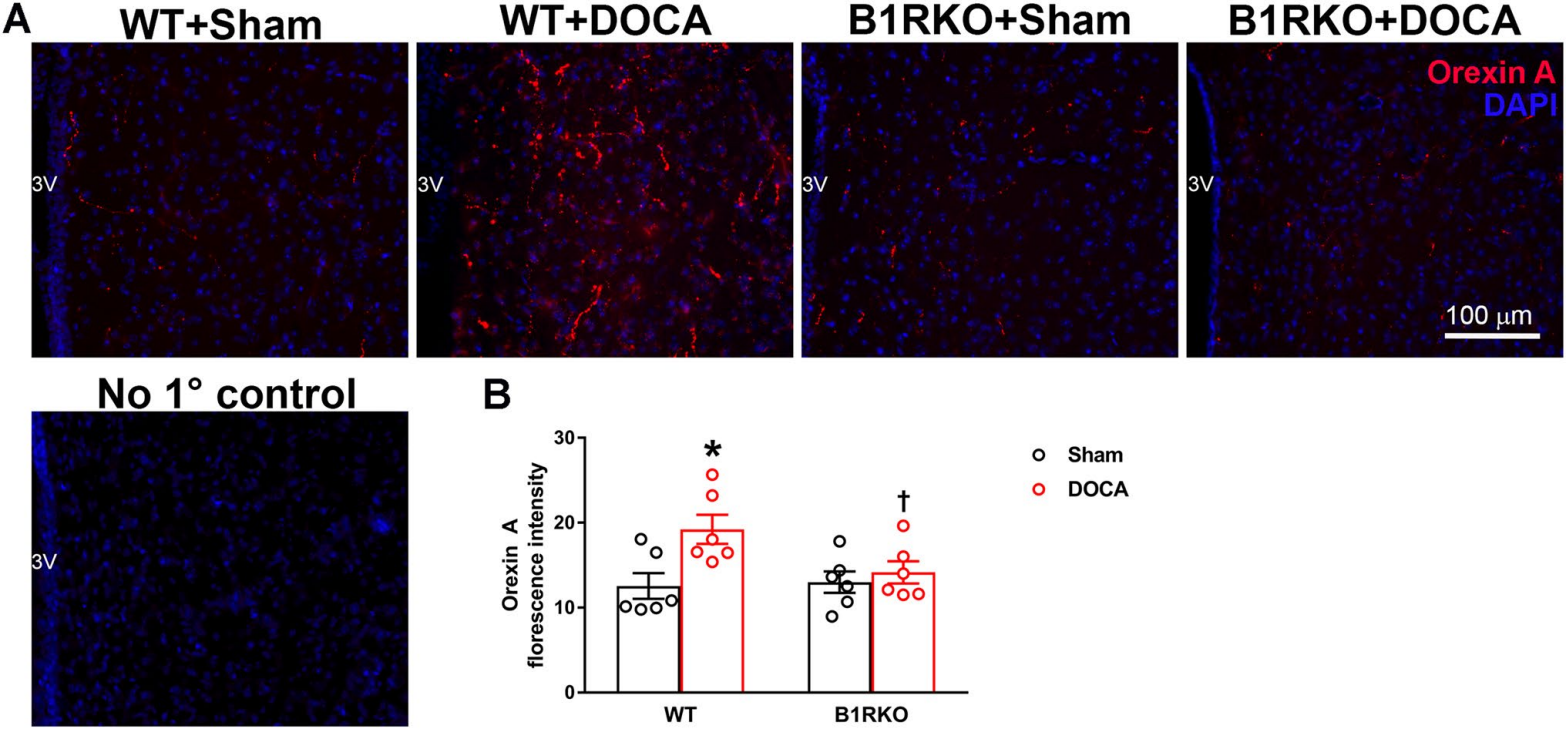
Orexin A protein expression and DOCA-salt-induced hypertension. (**A**) Immunofluorescence staining and (**B**) quantification reveals that DOCA-salt treatment significantly increased orexin A protein expression within the hypothalamic paraventricular nucleus of mouse brain. This increase in orexin A protein expression was attenuated in B1RKO mice with DOCA-salt hypertension suggesting that B1R knockdown reduces the activation of orexin system. Statistical significance: Two-way ANOVA followed by Tukey’s multiple comparisons test. *p < 0.05 versus WT + Sham; ^†^ < 0.05 versus WT + DOCA; n = 6/group. 3 V: third ventricle.

In WT mice, DOCA-salt-treatment increased (*P* < 0.05) orexin A protein expression in the hypothalamic paraventricular nucleus (PVN) (Fig. 1). However, similarly treated B1RKO mice exhibited lower orexin A protein expression (Fig. 1B, *P* < 0.01). This attenuation suggests that the genetic B1R knockdown reduces the activation of the orexin system in DOCA-salt treated mice.

### B1R knockdown mitigates upregulation of the PVN pro-hypertensive orexin receptors

**Figure 2:**
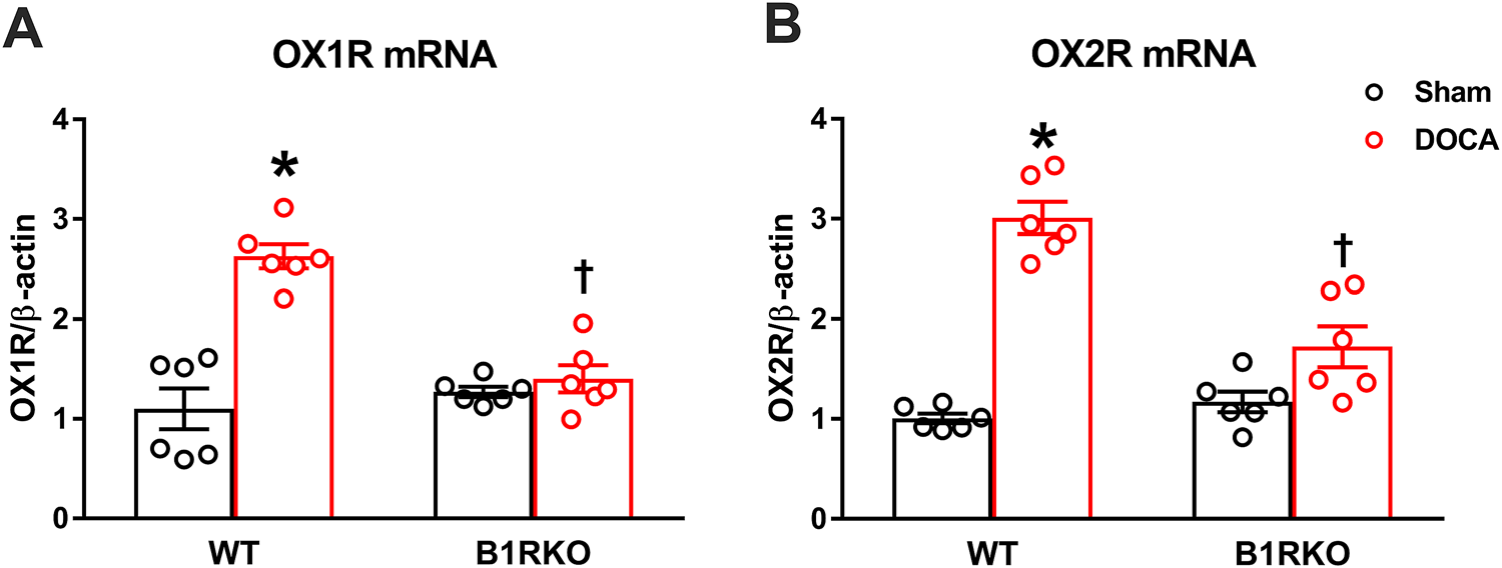
Gene expression of orexin receptors. DOCA-salt treatment significantly increased (**A**) OX1R and (**B**) OX2R mRNA in the brain hypothalamic PVN of WT mice, which was prevented in B1RKO mice with DOCA-salt hypertension. Statistical significance: Two-way ANOVA followed by Tukey’s multiple comparisons test. *p < 0.05 versus WT + Sham; ^†^*p* < 0.05 versus WT + DOCA; n = 6/group.

**Figure 3:**
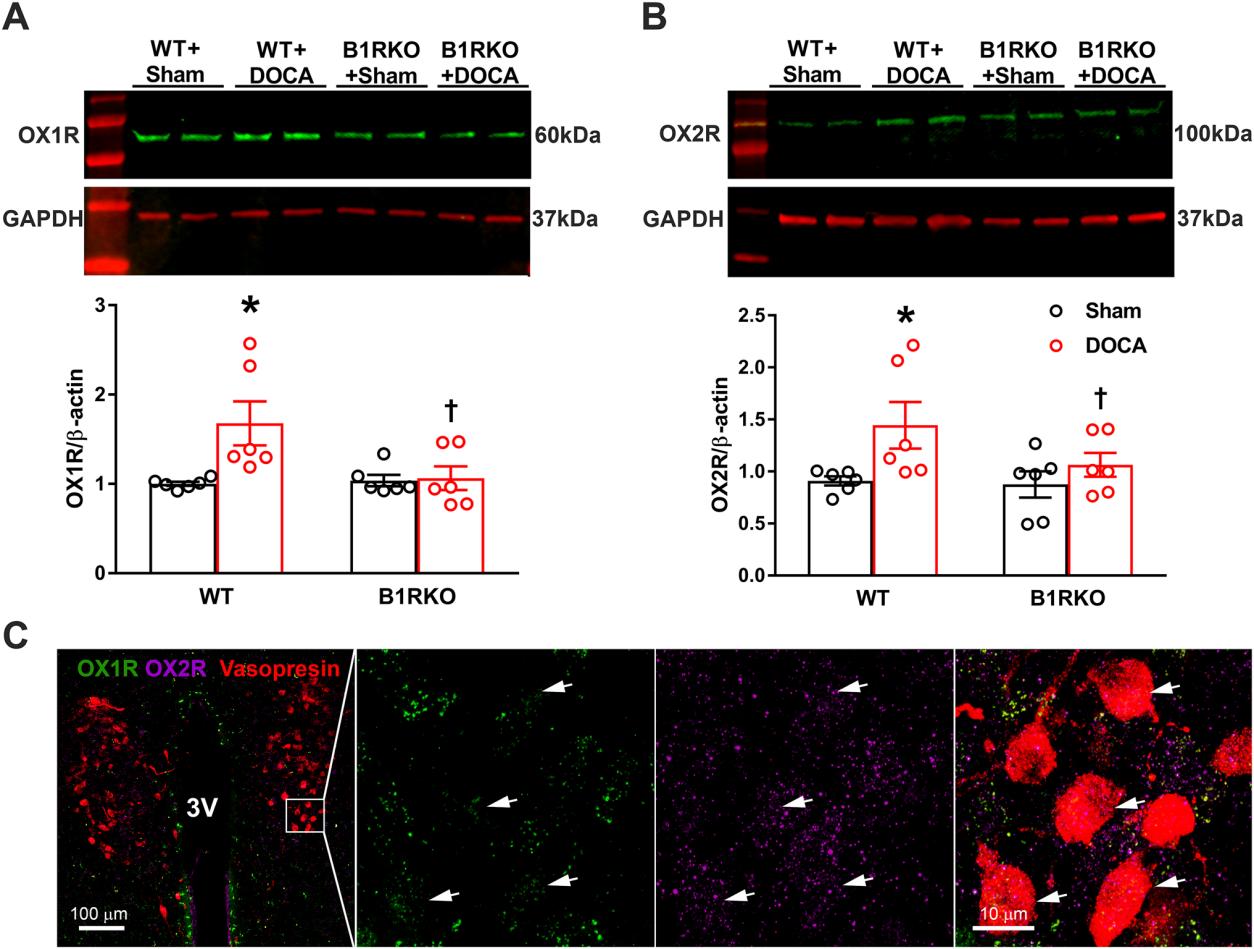
Orexin protein expression of orexin receptors within PVN. DOCA-salt treatment significantly increased (**A**) OX1R and (**B**) OX2R protein expression in the brain hypothalamic PVN of WT mice, which was prevented in B1RKO mice with DOCA-salt hypertension. Statistical significance: Two-way ANOVA followed by Tukey’s multiple comparisons test. *p < 0.05 versus WT + Sham; ^†^*p* < 0.05 versus WT + DOCA; n = 6/group. (**C**) Triple immunofluorescence staining of OXR1 (green) and OXR2 (magenta) along with neuronal anatomical marker vasopressin (red) reveals OX1R and OX2R staining co-localized with PVN neurons as pointed by arrows.

mRNA levels of both orexin receptors were upregulated (*P* < 0.05) in the PVN of DOCA-salt treated hypertensive WT mice when compared with sham-treated controls. This increase in mRNA expression was blunted in DOCA-salt treated B1RKO mice (Fig. 2A,B, *P* < 0.01). We further examined orexin receptor protein expression in both WT and B1RKO mice using hypothalamic PVN punches. DOCA-salt treatment increased expression of both orexin receptors OX1R and OX2R in the hypothalamic PVN of WT mice. However, this upregulation of the orexin receptors was attenuated (*P* < 0.05) in the B1RKO mice treated with DOCA-salt (Fig. 3A-B). Collectively, these data infers that the orexin system is upregulated in hypertension via B1R and that the knockdown of B1R reduces the activation of the orexin system. Triple immunolabelling of orexin receptors with vasopressin, a neuronal anatomical marker, showed co-localization of receptor immunoreactivity within neurons (Fig. 3C), suggesting neuronal orexin receptors overexpression during DOCA-salt induced hypertension (Supplementary Figure S1).

### B1R knockdown prevents DOCA-salt-mediated increase in plasma AVP

**Figure 4:**
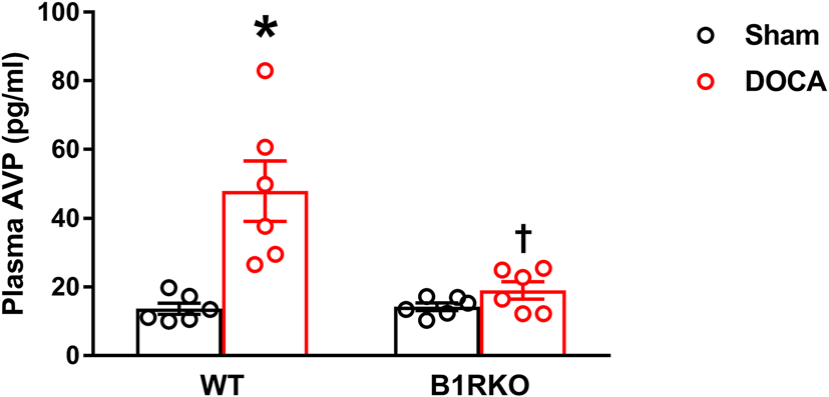
Plasma arginine vasopressin (AVP) expression in DOCA-salt hypertension. Three weeks of DOCA-salt treatment significantly increased plasma AVP levels in wild-type (WT) mice. This DOCA-salt-induced increase in plasma AVP was prevented in kinin B1 receptor knockout (B1RKO) mice with DOCA-salt treatment. Statistical significance: Two-way ANOVA followed by Tukey’s multiple comparisons test. *p < 0.05 versus WT + Sham; †, p < 0.05 versus WT + DOCA; n = 6/group.

A link exists between increase in plasma AVP and a hyperactive orexin system in hypertension^12^. Therefore, we measured the plasma AVP levels after 3 weeks of DOCA-salt treatment (Fig. 4). In DOCA-salt treated WT mice, plasma AVP was higher (*P* < 0.01) than sham operated controls. However, this DOCA-salt induced increase in the plasma AVP was blunted in B1RKO mice.

### B1R mediates orexin system hyperactivity in the primary hypothalamic neurons

**Figure 5:**
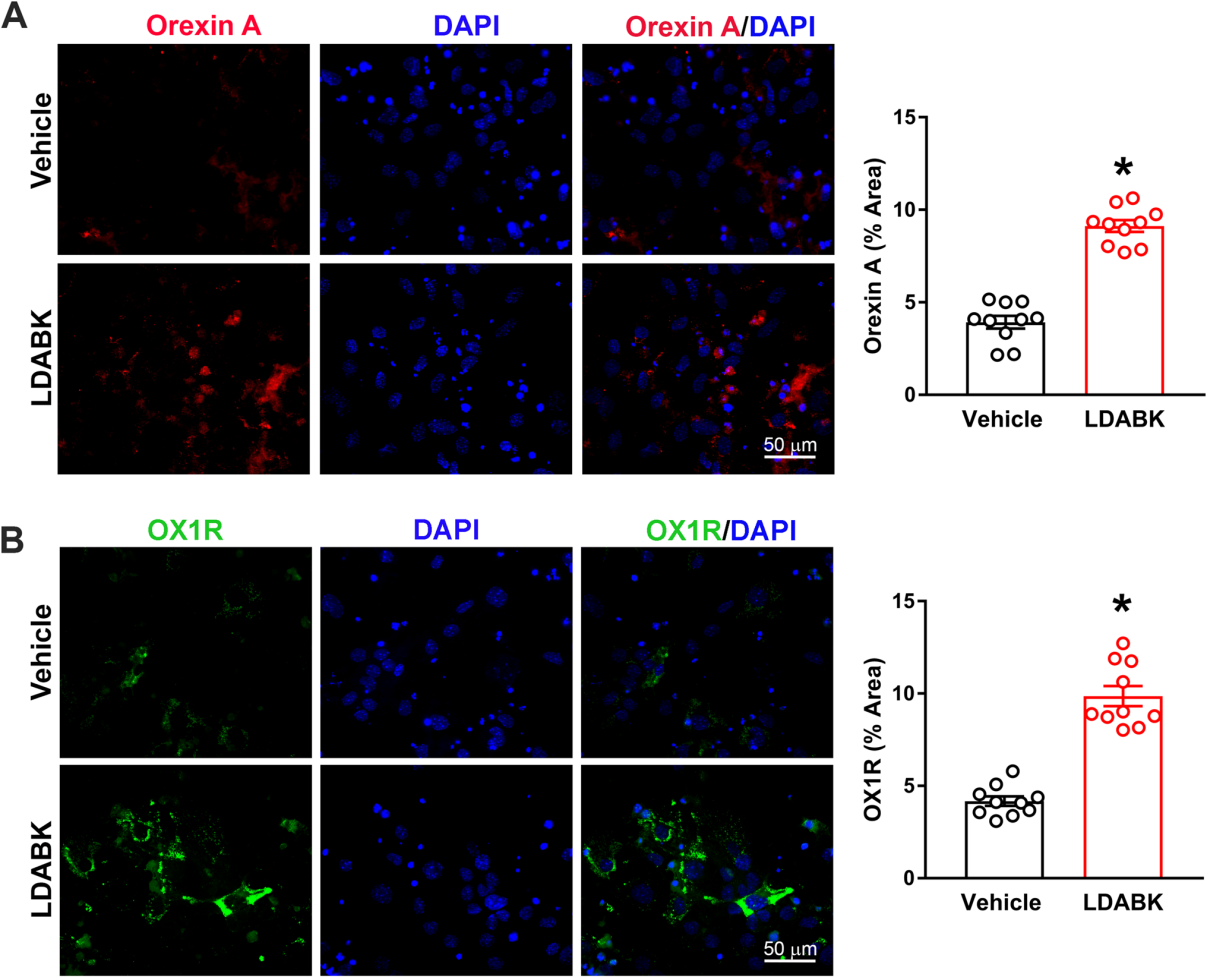
LDABK-mediated orexin A and orexin 1 receptor (OX1R) upregulation in primary hypothalamic neurons. Immunofluorescence staining and corresponding quantification data revealed that primary hypothalamic neurons stimulated with Lys-des-Arg-BK^9^, (LDABK, 300 nM) for 24 h showed an increase in orexin A (**A**) and OX1R (**B**) expression, indicating B1R activation leads to an upregulation in orexin system signaling. Statistical significance: Two-tailed *t* test. *p < 0.05 versus Vehicle; n = 10 images/group.

**Figure 6:**
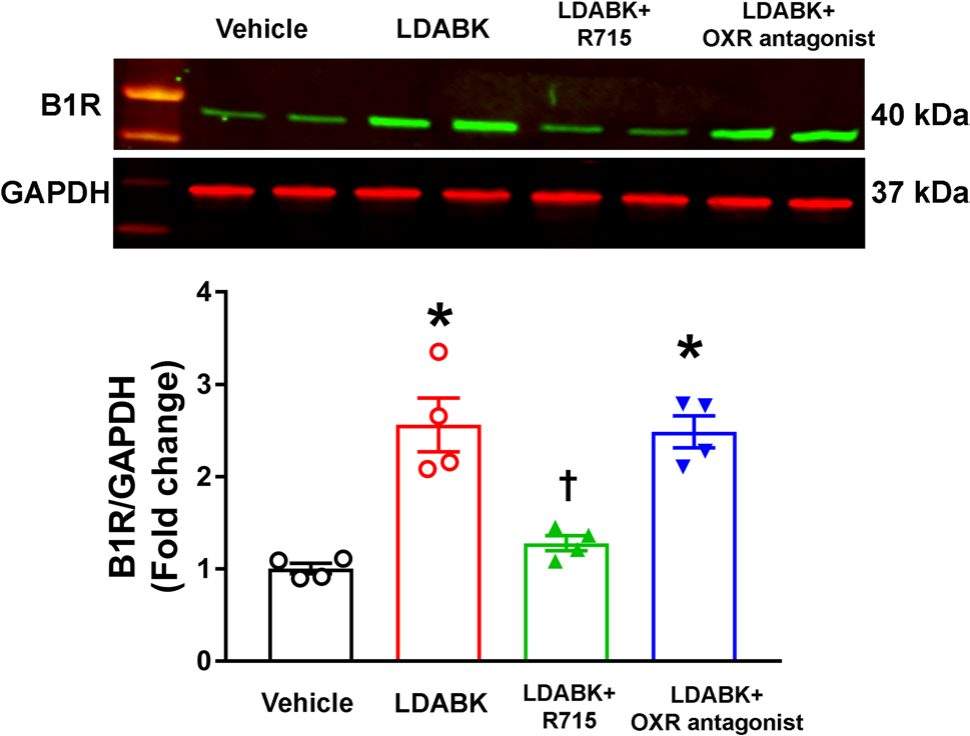
Orexin receptor antagonist fails to prevent B1R activation. Primary hypothalamic neurons stimulated with specific B1R agonist LDABK (300 nM) showed significant increase in B1R protein expression as measured and quantified by western blot analysis. Pretreatment with R715, a B1R specific antagonist, blunted this upregulation of B1R protein expression by LDABK. However, pretreatment with a dual orexin receptor antagonist failed to prevent LDABK-induced B1R upregulation. One-way ANOVA followed by Tukey’s multiple comparisons test. *p < 0.05 versus Vehicle; ^†^*p* < 0.05 versus LDABK; n = 4/group.

To discern the role of B1R in local orexin system activity, mouse primary hypothalamic neuronal cultures were used. B1R activation by its selective agonist, Lys-des-Arg-BK (LDABK)^9^, increased the expression (immunofluorescence) of orexin A (Fig. 5A) and OX1R (Fig. 5B). LDABK also increased the B1R protein expression (western), and this increase was abrogated by pre-treatment with the B1R antagonist R715 (Fig. 6). Interestingly, pre-treatment of primary neurons with dual orexin receptor (OX1R and OX2R) antagonist, ACT 462,206 did not prevent the LDABK-evoked increase in B1R expression (Fig. 6). These data clearly suggest that orexin system is downstream to that of B1R activation and that the blockade of B1R can prevent the activation of orexin system.

## Discussion

The overall findings of the present study are (1) genetic kinin B1R knockdown precludes overactivation of the PVN orexin system in a neurogenic hypertension mouse model; (2) elevation in plasma arginine vasopressin (AVP), which occurs in hypertensive mouse models, is attenuated in the B1R knockout mouse; (3) in primary neuronal culture, evidence suggests that neuronal B1R serves as an upstream activator of the orexin system. This investigative study offers novel insight into the critical signaling role of the kinin B1R in activating the orexin system and subsequent elevation of AVP in a mouse model of neurogenic hypertension.

Orexin is capable of increasing blood pressure, heart rate, and sympathetic nerve activity^28^ and plays a pivotal role in the development of neurogenic hypertension^6^. While other studies, including ours, implicated the PVN kinin B1R in neurogenic hypertension^15,16^, this is the first study that has dealt with the role of B1R in the modulation of the orexin system in the context of hypertension. Previous studies from our lab supported a causal role for B1R overexpression in DOCA-salt-induced hypertension because the hypertension was attenuated in the B1R-deficient mice^15,27^. The present study confirms these findings and demonstrates, for the first time, that PVN B1R overexpression was associated with local upregulation of the orexin system in wild type DOCA-salt-treated mice and both responses were mitigated in the B1R knockout mouse. Recent studies linked PVN orexin overactivity to elevated plasma AVP and blood pressure in a DOCA-salt rat model because orexin knockdown attenuated the pressor response^12^, which aligns with attenuation in plasma AVP levels in B1R-deficient DOCA-salt-treated mice in this study.

The low-renin DOCA-salt hypertension model is clinically relevant to patient populations with similar characteristics, such as African American patients and patients with type 2 diabetes mellitus-patient populations^15^ that have a higher incidence of resistant hypertension^2^. Unlike high renin hypertension models, the DOCA-salt hypertension, which is primarily driven by alterations in central mechanisms that regulate blood pressure, is well accepted as a model of neurogenic hypertension^29^. This study on kinin B1R reveals the importance of this target for the potential development of novel and targeted centrally acting pharmacological therapeutics for the treatment of neurogenic hypertension. Further, neuroinflammation and proinflammatory cytokine activity is augmented in the PVN of an animal model of salt-induced neurogenic hypertension^12^, and the kinin B1R appears to play a significant role as a potential mediator for neuroinflammation^16^. The overstimulation of proinflammatory pathways in the brain are well established as a primary contributing factor to initiating and maintaining neurogenic hypertension^30^, and neuroinflammation is capable of inducing cardiac autonomic dysregulation in male^31^ and female^32^ rats. Clinically, autonomic dysregulation predisposes patients to cardiac dysfunction^33^ and hypertension^34^. Further, many cases of resistant hypertension have a strong autonomic component, with increased sympathetic activity, and fall into the category of neurogenic hypertension^30^. These cases respond poorly to the peripheral RAS inhibitors that are classically the first line for the treatment of hypertension and may explain why the incidence of refractory hypertension is so high. However, currently available centrally acting sympatholytics have limited use due to their undesirable adverse effects.

**Figure 7:**
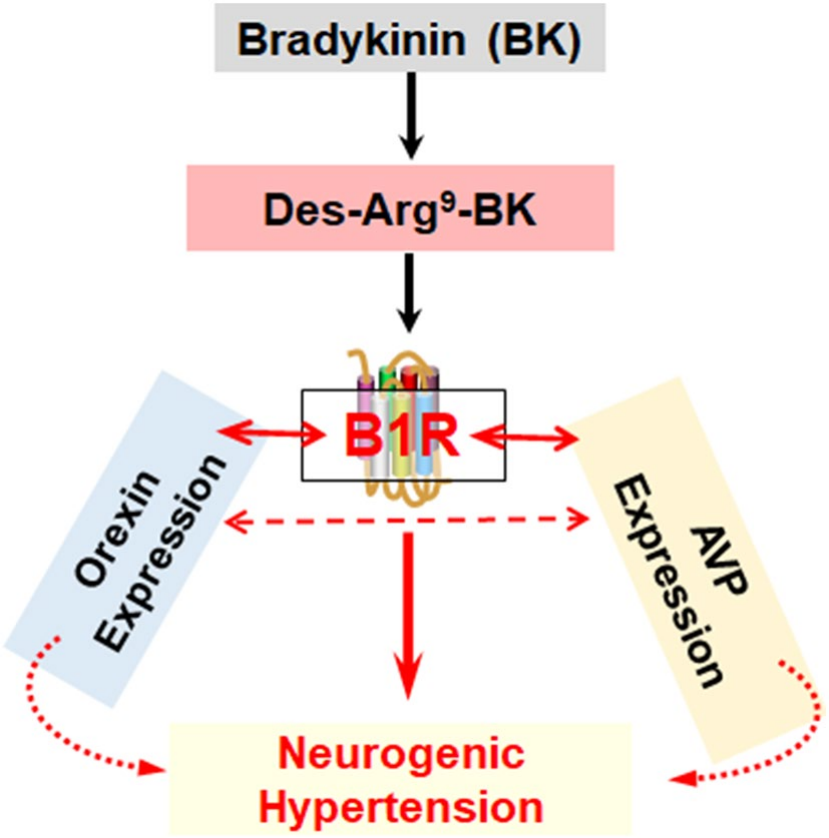
Kinin B1R mediates the activation of orexin system and hypertension. Bradykinin metabolites or kinin B1 receptor (B1R) agonists activate the B1R in the brain, which in turn modulates the expression of both orexin system and arginine vasopressin (AVP). This dual activation and increase of orexin and AVP contribute to the development and sustainment of neurogenic hypertension.

Our studies have clearly shown that B1R deletion attenuates the elevations in orexin A and orexin receptors expression in the PVN, as well as plasma AVP levels in DOCA-salt-treated mice^12^. While these findings yielded mechanistic insight into the role of PVN B1R in enhancing these two key mediators of neurogenic hypertension^12^, it was important to obtain pharmacologic evidence that undoubtedly supports this link. We show that pharmacologic activation of kinin B1R increases orexin system activity in primary neuronal culture, suggesting a link between B1R and orexin system, which contributes to neurogenic hypertensive state as discussed above. Based on these findings, we propose the signaling modulation mechanism shown in Fig. 7. Metabolites of bradykinin activate the kinin B1R, which in turn enhances orexin signaling and subsequently plasma arginine vasopressin (AVP). This signaling cascade contributes to the development and sustainment of neurogenic hypertension. It is noteworthy that this study did not deal with orexin B because via activation of its orexin 2 receptors, it causes anxietolytic effects along with decreased sympathetic activity^35^.

Intriguingly, once an agonist activates B1R, it does not appear to be internalized or desensitized, which indicates B1R is well situated to mediate the chronic actions of the orexin system and represents a novel therapeutic target for chronic inflammatory disease^15,36^. Additionally, once B1R is activated in a hypertensive state, the B1R endogenous agonist is able to upregulate the expression of its own receptor via the activation of the transcription factor NF-κB^37^. Given that B1R expression is upregulated in conditions of chronic stress and inflammation^38^, this suggests the creation of a detrimental cycle where endogenous activation leads to lasting effects. Additionally, B1R activation influences immune responses by modulating various immune cells and cytokine production^16,38^. This may help explain why the orexin A system contributes to adverse cardiovascular responses following acute and chronic stress^4,5^ and shows increased sympathetic activity as a main trigger and driver of essential hypertension^4^. Pharmacological blockade of kinin B1R could prove to be a valuable new tool in the control of neurogenic hypertension, particularly in patients with refractory hypertension by decreasing circulating AVP, downregulating the orexin A system, and decreasing neuroinflammation. The current study suffers some limitations. Functional studies are needed to evaluate the extent of the orexin system's contribution to sympathetic activity, increased AVP, and hypertension. Additionally, it is necessary to point out that our in vivo studies did not determine whether these signaling pathways are activated within a single cell type or whether the activation occurs simultaneously within multiple cell types. Our in vitro data support the hypothesis that B1R activates orexin A system in primary neuron cultures. However, further studies are needed to elucidate the signaling pathways within specific cell types of the PVN or other regions of the brain that are involved in blood pressure control.

The elevated expression and activation of B1R is observed in numerous diseases associated with inflammation, including hypertension, heart failure, stroke, atherosclerosis, obesity, diabetes, asthma, traumatic brain injury, and neurodegenerative disorders^16^. The present study clearly supports the hypothesis that kinin B1R plays a central role in mediating orexin A system hyperactivity in neurogenic hypertension. Our studies utilized both a genetic knockout animal model and pharmacological activation of the B1R in primary neuronal culture to highlight the importance of B1R in exacerbating the orexin system activity in neurogenic hypertension, supporting B1R as a viable target for antihypertensive drug development. Overall, the study adds new knowledge on PVN B1R signaling in hypertension.

The results of the present study demonstrate that deletion of kinin B1R mitigates the increased activity of the central orexinergic system and elevated plasma AVP, well-recognized mediators of the DOCA-salt hypertension. Ex vivo findings in PVN tissues from DOCA-salt treated wild-type and B1R deficient mice and pharmacologic studies with B1R agonist and antagonist as well as orexin receptor antagonists in neuronal culture support the premise that B1R serves as an upstream activator of the pro-hypertensive orexin A system. Equally important, the findings identify the B1R blockade as a novel central therapeutic modality, particularly in resistant hypertension. Further studies on the role B1R in the modulation of hypertension and neuroinflammation are needed to substantiate these findings and to understand this complex signaling cascade. These future investigations will also provide foundational knowledge and provide insights into the development of future treatments for clinically treating neurogenic hypertension.

## Supplementary Information

Supplementary Information.
