# Supplementary material for: Hypothalamic kinin B1 receptor mediates orexin system hyperactivity in neurogenic hypertension
Source: Sci Rep. 2021 Oct 26;11:21050. doi: 10.1038/s41598-021-00522-0 (PMC8548389; doi:10.1038/s41598-021-00522-0)
Supplement: Supplementary file 1 — Supplementary Information. [file 41598_2021_522_MOESM1_ESM.pdf]

Hypothalamic Kinin B1 Receptor Mediates Orexin System Hyperactivity in Neurogenic Hypertension

Rohan Umesh Parekh[1], Acacia White[1], Korin E Leffler[1], Vinicia C. Biancardi[2], Jeffrey B. Eells[3], Abdel A. Abdel-Rahman[1], and Srinivas Sriramula[1]*

[1]Department of Pharmacology and Toxicology, Brody School of Medicine at East Carolina University, Greenville, NC 27834, USA. [2]Department of Anatomy, Physiology & Pharmacology, College of Veterinary Medicine, Auburn University, Auburn, AL, USA; Center for Neuroscience Initiative, Auburn University, Auburn, AL, USA. [3]Department of Anatomy and Cell Biology, Brody School of Medicine at East Carolina University, Greenville, NC 27834, USA.

*Corresponding Author:

Dr. Srinivas Sriramula, PhD
Department of Pharmacology and Toxicology,
Brody School of Medicine, East Carolina University
600 Moye Blvd,
Greenville, NC 27834, USA.
Phone number: +1.252.744.2746
Fax number: +1.252.744.3203
E-mail: sriramulas17@ecu.edu

Supplementary Figure S1

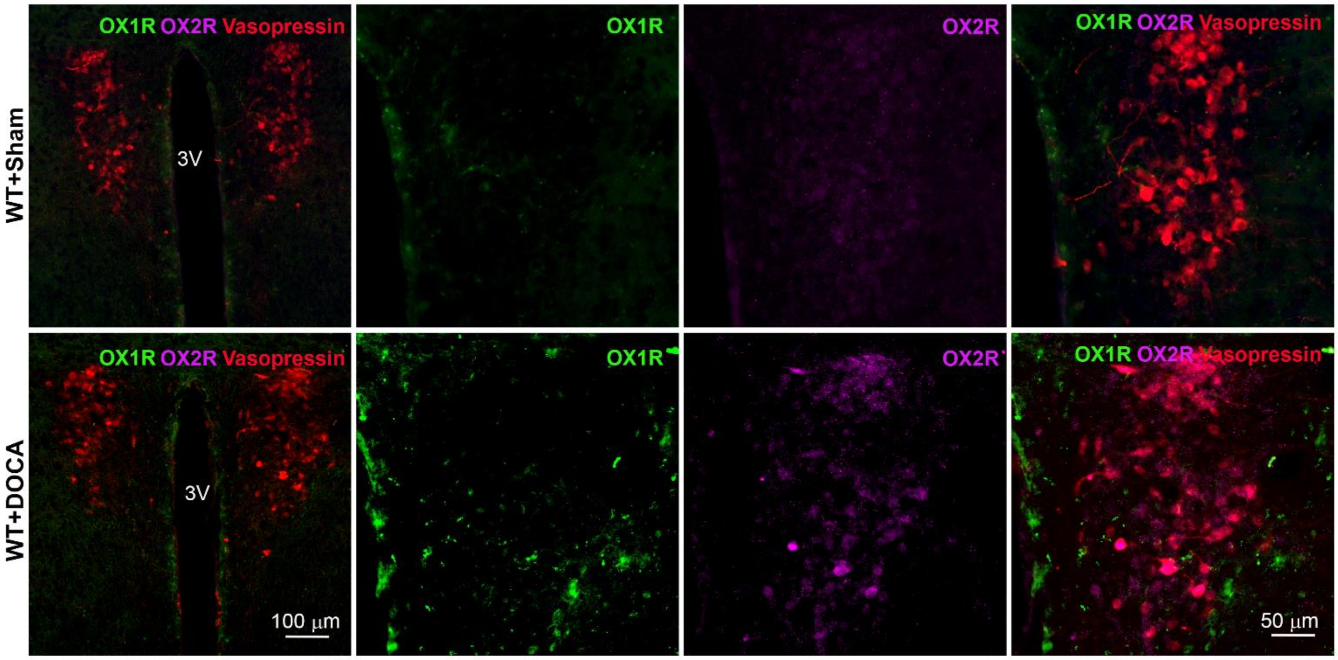

Figure S1. Triple immunofluorescence staining of OXR1 (green) and OXR2 (magenta) along with anatomical marker vasopressin (red) reveals increased OX1R and OX2R staining in the PVN neurons during DOCA-salt induced hypertension**.** 3V: third ventricle.

Supplementary Figure S2

OX1R:

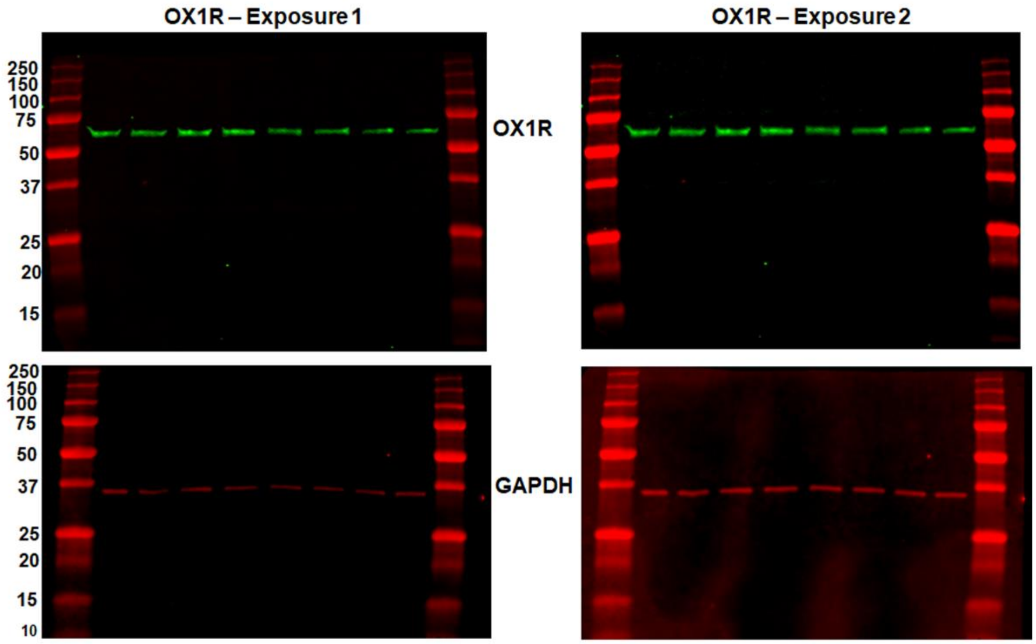

OX2R:

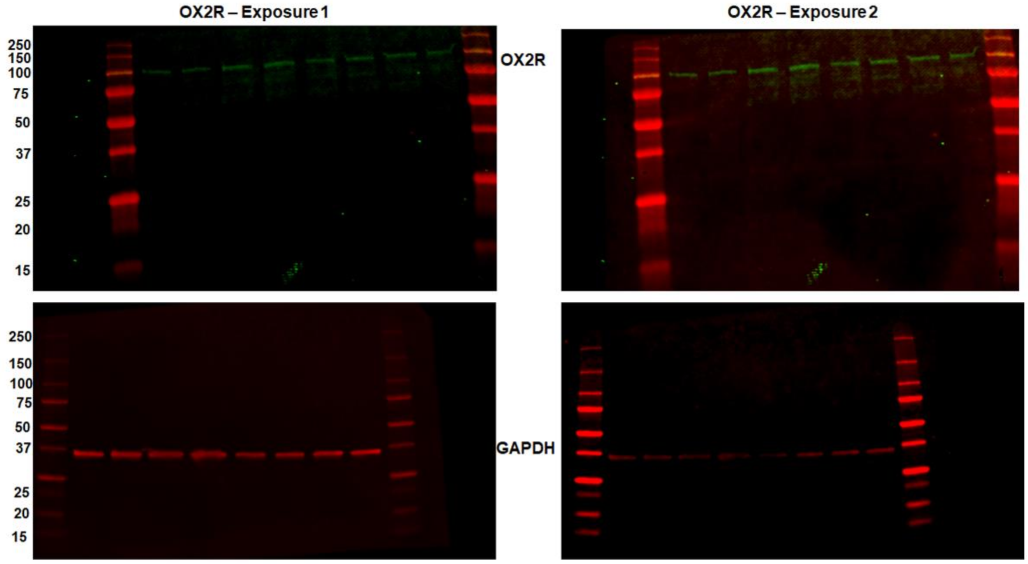

Figure S2. Full length western Blots used for the images in the Figure 3.

Supplementary Figure S3

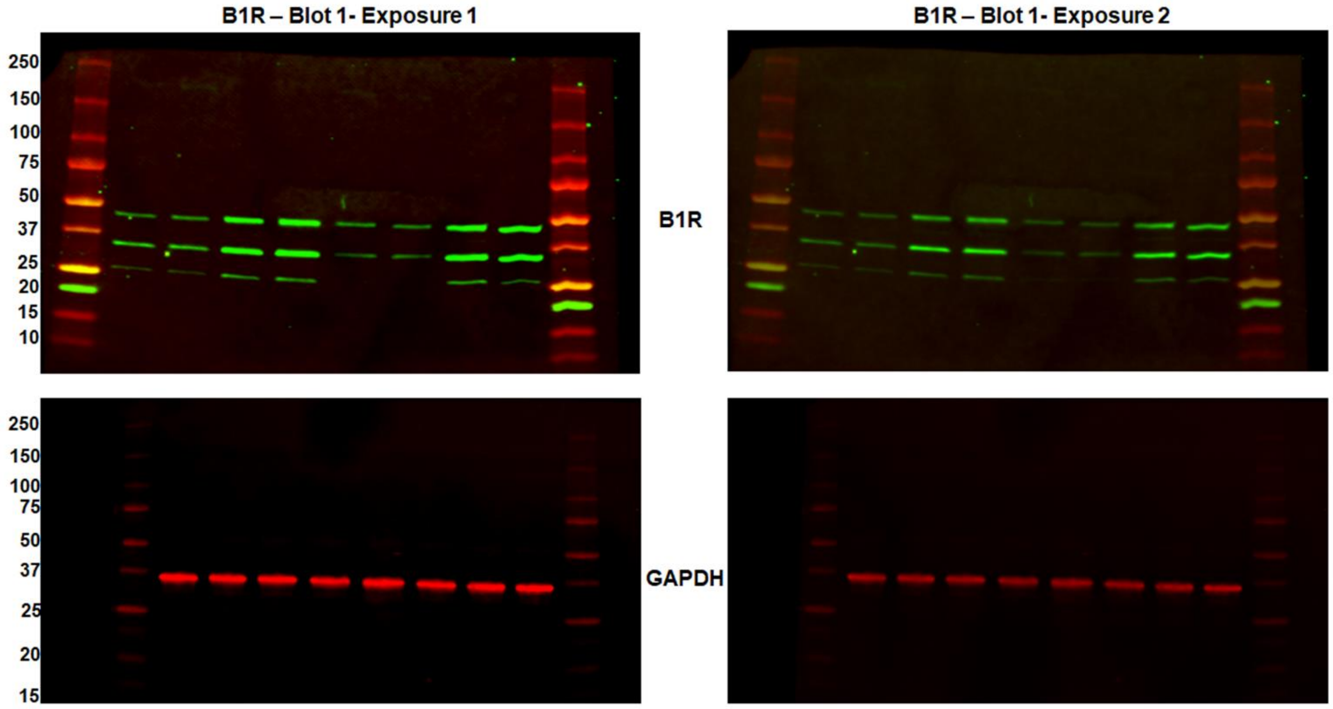

Figure S3. Full length western Blots used for the images in the Figure 6.

Supplementary Figure S4. B1R antibody validation using knockout neurons.

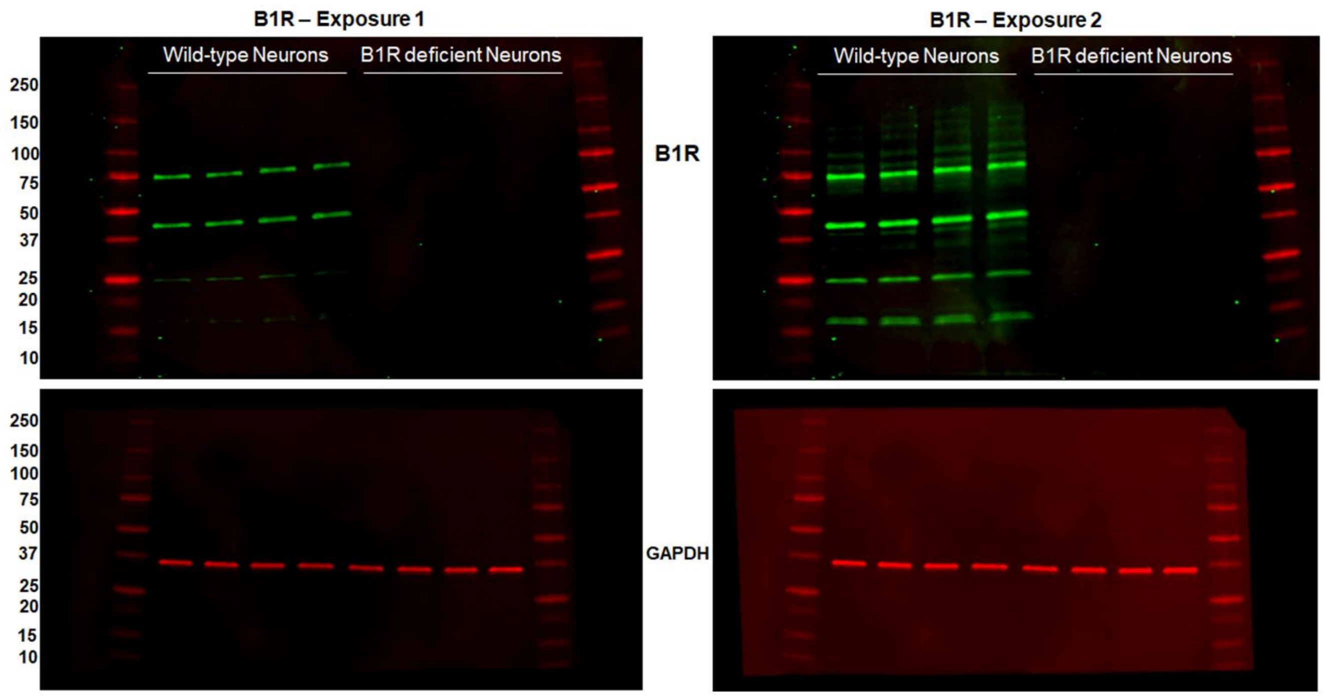

Figure S4. B1R antibody validation using B1R deficient neurons isolated from B1R knockout mice. Western blot analysis of primary hypothalamic neurons isolated from wild-type control and B1R knockout mice using antibodies against B1R (1:250, #ABR-011, Alomone labs) and GAPDH (1:1000, #MAB374, Millipore Sigma) and visualized using Odyssey CLx imaging system (Licor). The two different exposures of the blots were shown.
